# The Controversial Push for New Brain and Neurorights

**DOI:** 10.2196/72270

**Published:** 2025-02-25

**Authors:** Simon Spichak

## Abstract

Advances in neurotechnology that can measure or influence brain activity are prompting debate about whether existing privacy and human rights protections are sufficient. In this News and Perspectives article, JMIR Correspondent Simon Spichak reports on the push for “neurorights”, highlighting a landmark Chilean case and emerging legislation.

In August 2023, the Supreme Court of Chile issued a unanimous decision ordering the US-based brain-computer interface company Emotiv to erase the brain data it had collected on a former Chilean Senator [1].

The results of the court case were reported as a landmark decision for “neurorights”—enacting the country’s recent constitutional provision to protect people’s brain data and mental privacy from being sold or manipulated by neurotechnology. Rafael Yuste, a codirector of Columbia University’s Neurotechnology Center and cofounder of the Neurorights Foundation, took part in crafting the provision.

The Neurorights Foundation is instrumental in the push for new human rights and legislation to protect people’s brains from neurotechnology.

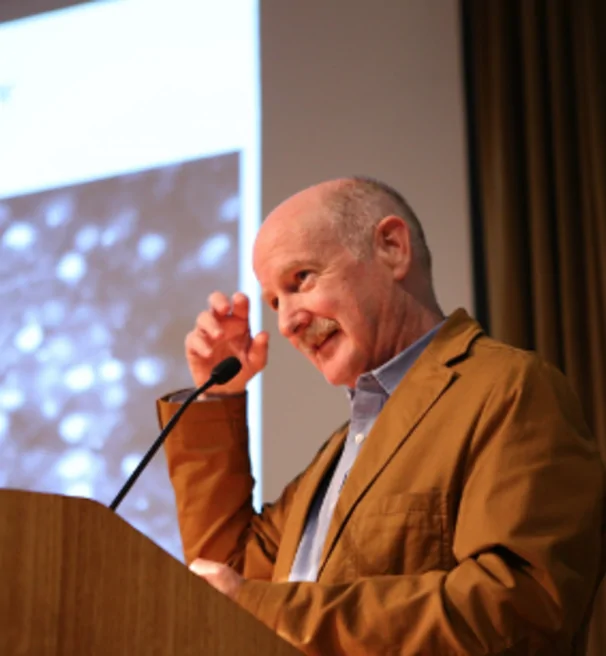

Rafael Yuste delivering a lecture in Chile.

However, these efforts have proven controversial among scientists, legal scholars, and advocacy groups [2-4]. Yuste told JMIR Publications (JMIR) that some of the criticism is “poorly informed” and that neurorights are not adequately covered by existing rights or data privacy legislation. He added that some of the critics may receive funding from the industry.

(The experts who spoke with JMIR disclosed that they had not received funding from neurotechnology companies.)

J Carlos Lara Gálvez, executive director of Derechos Digitales, a nonprofit organization based in Chile that advocates for digital rights across Latin America, told JMIR that legal experts in the country are split on the neurorights provision, with some skeptical that “such legal and constitutional changes are needed at all.”

Danielle Zaror Miralles, a law and technology professor at the University of Chile Law School, explained to JMIR that the constitutional provisions are based on the colonial idea that “there were no legal protections” for brain data in Chile before this legislation.

## Why are some researchers advocating for “neurorights”?

Neurotechnology encompasses any device that can directly read, measure, or manipulate brain data. Some invasive forms of neurotechnology, like deep brain stimulation (DBS), are implanted directly into the brain to treat the symptoms of Parkinson disease, depression, obsessive-compulsive disorder, and other diseases [5].

These forms of neurotechnology are regulated as medical devices, while consumer-facing devices are largely unregulated in countries like the United States.

“I became very concerned with this [neurorights] through our own experiments with mice,” Yuste told JMIR. “About a decade ago, we were able to decode the activity of the visual cortex and manipulate it selectively using laser-based technology.”

While invasive brain implants or “mind-reading” devices aren’t hitting the consumer market anytime soon, many noninvasive consumer electronics—like Muse or Emotiv—are already on the market. These devices measure electroencephalographic (EEG) brain data to help measure and potentially improve sleep and mental health. Researchers are also developing “mind-controlled” drones using consumer-grade EEG headsets [6]. In 2023, Apple also received approval on its patent for AirPod-like earbuds with built-in electrodes that could theoretically measure and collect EEGs [7].

Marcello Ienca, a professor who studies the ethics of artificial intelligence (AI) and neuroscience at the Technical University of Munich and the Swiss Federal Institute of Technology Lausanne, also believes this raises new problems. “The potential for intervening into brain function, either by recording activity from the brain, or for writing into the brain using neurostimulation techniques, opens challenges that are substantial from a human rights perspective, and that can be well understood as human rights challenges,” he told JMIR.

In response to the development of this technology, Yuste and other experts believe that new “neurorights” relating to brain data are needed [8,9].

1. **The right to mental privacy.** To prevent neural activity from being deciphered without consent.
2. **The right to mental identity.** To protect against technology altering the self, consciousness, or personality.
3. **The right to free will.** To prevent neurotechnology from tampering with decision-making abilities.
4. **Fair access to mental augmentation.**
5. **Protection from bias embedded in neurotechnology algorithms.**

Other experts are more skeptical of neurorights. “While there is rough consensus on the potential risks of certain technologies that interact with the human body or the brain, there is no consensus on the notion that those risks are certain or well-defined, that those risks are current and actual instead of potential and speculative,” Gálvez said. “Some of the most publicly visible support comes accompanied by highly exaggerated claims about the capabilities of these technologies.”

Gálvez is concerned that the debate on neurorights displaces attention and discussion from other fundamental rights and data protection regulations.

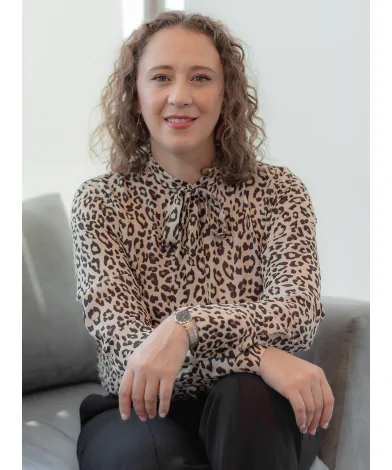

Danielle Zaror Miralles, law and technology professor at the University of Chile Law School.

## So, are new human rights needed to protect the brain?

The Neurorights Foundation analyzed seven existing international human rights treaties and determined that they included insufficient protections for brain data, concluding that amendments or new rights were needed to protect brain data [10].

Nita Farahany, a legal scholar and bioscience professor at Duke University who wrote *The Battle for Your Brain*, also thinks that new rights are needed. She argues for a broader approach through the lens of “cognitive liberty,” which would protect self-determination, freedom of thought, and mental privacy. Farahany told JMIR that cognitive liberty could provide broad protections against technologies that can predict cognitive states, even if they don’t directly measure neural data.

“If the biometric data is being used to make inferences about a person's mental state, it deserves a higher level of protection,” she said.

Ienca told JMIR that he’s agnostic about the right approach to protecting these rights—whether reinterpreting existing rights or introducing new ones.

Several legal experts told JMIR they don’t think this new framework is needed.

Susie Alegre, a human rights lawyer, author of the book *Freedom to Think,* and a senior fellow at the Center for International Governance Innovation, told JMIR that adding new rights or amending existing ones doesn’t make sense. “The big problem is that when you say we need new rights, then you are firstly saying the rights we have don't cover this, and therefore, it's a kind of a free pass,” she said. “Fundamentally, I don’t understand advocating for new rights without testing the boundaries of existing rights in court.”

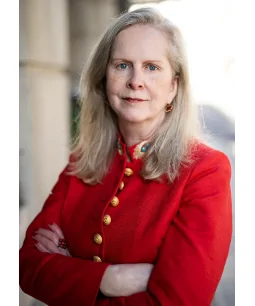

Susie Alegre is a human rights lawyer, author of Freedom to Think, and senior fellow at the Center for International Governance Innovation. Photo credit: Tugce Nelson.

Jan Cristoph Bublitz, associate junior professor of criminal law at the University of Hamburg, also argued against the proposal for neurorights in a 2022 paper in the journal *Neuroethics* [11]. “The proposal tends to promote rights inflationism, is tainted by neuroexceptionalism and neuroessentialism, and lacks grounding in relevant scholarship,” he argued.

In 2022, the UN Human Rights Council asked its Advisory Committee to study the issue [12].

Liz Throssell, a spokesperson from the UN Human Rights Office, said that the existing human rights framework does address the issues related to privacy and autonomy. Throssell told JMIR that there may be emerging issues outside the scope of legislation, but these will require further discussion.

“But the bottom line is that huge and potentially very consequential work needs to be done now in ensuring existing human rights standards are applied,” Throssel said, explaining that adding or amending human rights takes a lot of time. “What is important is that we focus on implementing the human rights standards we have at our disposal.”

## Legislating neurorights around the world

The Neurorights Foundation recently surveyed 30 user agreements from direct-to-consumer neurotechnology companies. Clicking “I agree” on 29 of these agreements would give the neurotechnology company any rights to the data, which would allow them to sell it to a third party. Yuste called these user agreements “predatory.”

“There are no rules; the company is taking everything,” he said. “That's why we're working with many countries and international organizations to protect brain data.” Ultimately, he believes that brain data should be protected as stringently as medical data.

Following the constitutional provisions in Chile, which Yuste and the Neurorights Foundation helped influence, other countries like Uruguay, Brazil, and Mexico have all either proposed or pushed through constitutional amendments that would provide similar protections.

However, experts, including Miralles, say these provisions are unnecessary as the constitutional protections in Latin America sufficiently encompass brain data and can restrict the use of medical neurotechnologies because of how the law is written [4].

Miralles and Pablo Contreras, a law professor at the Central University of Chile, argued that the neurorights provision was irrelevant to the Emotiv case, arguing that the company deleted the data in conjunction with existing data protection laws [13]. “Most constitutions on the continent recognize the right to privacy, and almost all countries have laws on personal data protection inspired by the GDPR,” she said. “Needless to say, the entire hypothesis of this legislation is based on a science fiction fantasy idea.”

Gálvez told JMIR that there were concerns the constitutional provision led to “altering the functions of public health authorities and the application of data protection law, introducing confusion to the legal landscape.”

Meanwhile, in the United States, the Neurorights Foundation also worked alongside the Colorado Medical Society to sponsor bill HB24-1058 to protect brain activity and brain data.

Other states have enacted broader laws to protect other types of biometric data that technology companies collect. Illinois’s Biometric Information Privacy Act, for example, requires private entities collecting biometric data to inform people about what kind of data is being collected, how it’s stored, and how it’s used.

“I think that's a good start, but I would go further than it does,” Farahany said, adding that cognitive biometric data should, for example, be kept on the device where it is collected and not sent to an external company.

In the European Union, Alegre explained that there are already laws and regulations protecting people’s data from exploitation, but the problem involves enforcing and regulating these rules, adding that “the biggest problem is it's very hard to take a tech company to court.”

*JMIR News & Perspectives articles are written by journalists. They are not peer-reviewed but rigorously fact-checked. We invite freelance journalists to pitch us original ideas.*
